# Spontaneous Calyceal Rupture Due to a 3-mm Obstructing Ureteric Stone: A Case Report

**DOI:** 10.7759/cureus.76999

**Published:** 2025-01-06

**Authors:** Syed Yousaf Khalid, Tauqir Aslam Waraich, Osman Muhammad, Shawgi Omer

**Affiliations:** 1 Cardiothoracic Surgery, St. James's Hospital, Dublin, IRL; 2 Department of Urology, Letterkenny University Hospital, Letterkenny, IRL

**Keywords:** calyceal rupture, radiology, renal calculi, urinoma, urolithiasis

## Abstract

Spontaneous rupture of the renal collecting system due to an obstructing ureteric stone is a rare, but significant complication. We present the case of a 27-year-old woman who presented with sudden, severe abdominal pain initially suspected to be of gynecological origin. Further investigation revealed a 3-mm obstructing stone at the vesicoureteric junction causing calyceal rupture. Rupture due to such small stones is rare and may be overlooked on non-contrast CT; however, the presence of perinephric edema or fluid should raise suspicion of this complication. The diagnosis was confirmed using contrast-enhanced CT, which demonstrated contrast extravasation. The patient was initially managed conservatively with analgesia, antibiotics, and alpha-blockers; however, persistent pain and the risk of worsening urinary extravasation necessitated cystoscopy and JJ stent placement. The postoperative recovery was favorable, and the patient was discharged on the second day. Follow-up ureteroscopy revealed no residual stones and the stent was successfully removed. This case highlights the importance of early diagnosis and timely urological intervention to prevent complications, such as urosepsis, urinoma, and acute kidney injury. While conservative management may suffice for small, passable stones, stenting is necessary in cases of obstructive uropathy, infection, or significant rupture. This report emphasizes the clinical presentation, diagnostic challenges, and management strategies of this rare but important condition. Prompt recognition and appropriate treatment are essential to achieve favorable outcomes.

## Introduction

Urolithiasis affects approximately 1 in 11 individuals during their lifetime, with men being twice as likely to experience it, compared to women [1]. Factors contributing to the formation of kidney stones include dehydration, hypercalciuria, hyperoxaluria, hyperuricosuria, and hypocitraturia [2]. It has various complications, including abscess formation, forniceal rupture, hydronephrosis, perinephric abscess, pyelonephritis, pyonephrosis (obstructive pyelonephritis), renal colic, renal failure with potential progression to kidney atrophy or end-stage kidney disease, sepsis or urosepsis, ureteral calculi causing colic, obstruction, pain, scarring, urinary extravasation, and formation of urinomas [2].

Spontaneous rupture of the urinary collecting system is a rare condition characterized by leakage of urine from the collecting system into the perirenal space or retroperitoneum, occurring in the absence of trauma or medical intervention involving the ureter. This rupture results from elevated intraluminal pressure, typically caused by urinary calculi, strictures, or external compression [3].

Here, we present a case of calyceal rupture resulting from a 3-mm obstructive stone located at the vesicoureteral junction (VUJ).

## Case presentation

**Figure 1:**
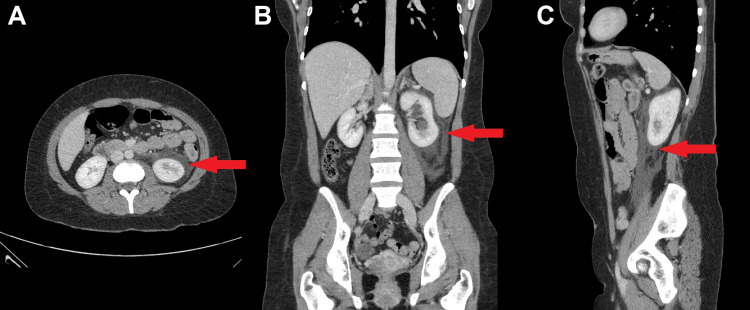
Axial (A), coronal (B), and sagittal (C) views of CT of the abdomen and pelvis with contrast showing left perinephric inflammatory changes and fluid tracking (arrows), suggestive of a urine leak extending down the retroperitoneum

**Figure 2:**
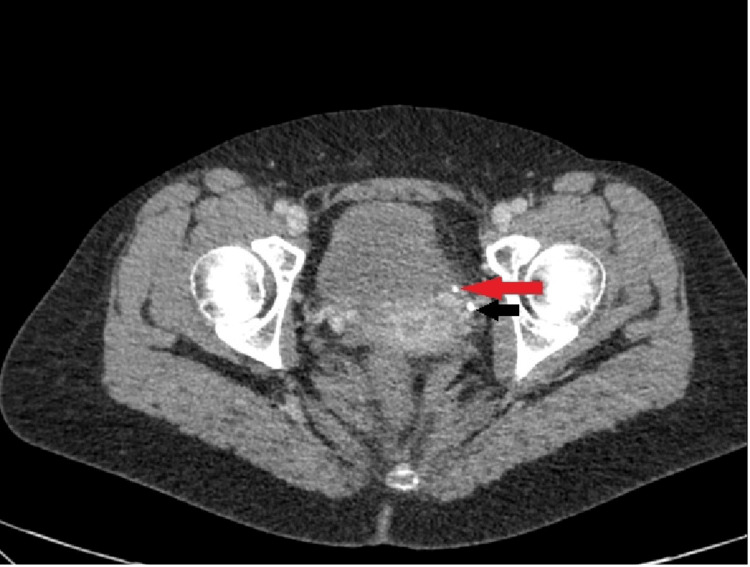
CT (axial view) of the pelvis showing the 3-mm obstructing stone at the left vesicoureteric junction  (red arrow), and associated inflammatory changes and a phlebolith (black arrow)

A 27-year-old woman presented to the gynecology department with acute abdominal pain that was more pronounced on the left side, accompanied by nausea and vomiting. She had a history of ovarian cysts, but exhibited no signs of peritonitis or fever. Renal function tests were normal, and the pregnancy test was negative. However, her C-reactive protein (CRP) level was elevated to 225 mg/L (reference range, <5 mg/L), and her white blood cell (WBC) count was 19 × 10^9^/L (reference range, 4-11 × 10^9^/L). Initially, rupture or torsion of the cyst was suspected, which was ruled out by a bedside ultrasound performed by the gynecology department. However, contrast-enhanced CT of the abdomen and pelvis revealed a 3-mm obstructed VUJ calculus, moderate hydronephrosis, and perinephric inflammatory changes with fluid extending into the retroperitoneum (Figures 1, 2). The patient was initially managed conservatively with analgesics, antibiotics, and alpha-blockers. Given the small size of the stone, there was a good chance that it would pass spontaneously, thus avoiding the risks associated with invasive procedures. Despite close monitoring, due to persistent pain and the risk of worsening urinary extravasation, she underwent cystoscopy and JJ stenting after 24 hours. The patient showed significant postoperative clinical improvement. Follow-up ultrasonography of the kidney showed no perirenal collection. The patient was discharged on postoperative day 2 with instructions for hydration, pain management, antibiotics, and follow-up for ureteroscopy.

Follow-up ureteroscopy was performed after eight weeks, and the JJ stent was removed. No residual stone was present.

## Discussion

Spontaneous rupture of the renal calyx, although uncommon, can occur because of various causes including ureteral stones, strictures, and tumors [3]. Our case illustrates rupture of the left renal calyx due to a 3-mm obstructing VUJ stone, leading to perirenal urine extravasation. Calyceal rupture is often a result of increased pressure in the urinary collecting system and serves as a protective mechanism to prevent further renal damage [4]. This pressure can lead to extravasation of urine into the perirenal space or retroperitoneum, as observed in our case.

Patients usually present with sudden onset of abdominal pain, often resembling renal colic, making the diagnosis challenging and frequently delayed. The severity of symptoms can vary widely, ranging from mild flank pain to severe acute abdominal pain, further complicating the diagnostic process [5,6].

Non-contrast-enhanced computed tomography (NCCT) is now the preferred diagnostic tool for acute flank pain, having supplanted intravenous urography (IVU). NCCT provides detailed information about stone location, size, and density. In cases in which stones are not present, it aids in identifying alternative causes of abdominal pain. When assessing patients with suspected acute urolithiasis, NCCT demonstrates superior accuracy compared to IVU or ultrasound [7]. Perinephric edema or fluid collection observed on the initial imaging suggests the possibility of calyceal rupture and should prompt further investigation. This diagnosis can be confirmed with a contrast-enhanced CT scan, which can detect contrast leakage and/or urinoma formation. A delayed-phase protocol on contrast-enhanced CT offers higher sensitivity as it highlights asymmetric delayed contrast excretion at the obstruction site. Additionally, it aids in distinguishing small kidney stones from vascular calcifications [8].

The management of calyceal rupture depends on the severity of symptoms and stone size. Conservative treatment, including hydration, analgesia, and medical expulsive therapy with alpha-blockers, is often effective for small passable stones. Approximately 98% of ureteric stones measuring less than 5 mm, 60% of those measuring 5-7 mm, and 39% of stones measuring >7 mm typically pass spontaneously within four weeks [9]. This case is notable because despite the small size of the stone (3 mm), it caused a significant obstruction. Medical expulsive therapy is recommended for small stones, as it is cost-effective and enhances the likelihood of spontaneous stone passage without the need for surgical intervention. Various medications can be utilized, including alpha-blockers, calcium channel blockers, and phosphodiesterase-5 inhibitors. Among these, tamsulosin has proven to be the most effective alpha-blocker, offering superior outcomes in terms of expulsion rate, reduced expulsion time, and overall safety [10]. However, intervention is necessary in cases with persistent symptoms, risk of infection, or worsening extravasation. In our case, the patient initially received conservative treatment but required cystoscopy and JJ stent placement due to persistent pain and the risk of complications [11,12]. This approach effectively relieved obstruction and prevented further damage.

Spinelli et al. conducted a study involving 1,629 patients presenting to the emergency department with renal colic, among whom 31 had spontaneous rupture of the upper urinary tract, similar to our patient’s condition [13]. The VUJ was identified as the most frequent location of obstructing stones, as in our case. Chaabouni et al. described a case comparable to ours, involving a 61-year-old woman who presented with lumbar pain accompanied by nausea and vomiting. CT revealed a calyceal rupture with urinoma formation caused by a 4 × 2 mm stone in the distal ureter. The stone was successfully removed, a double-J catheter was inserted, and antibiotic therapy was initiated [14].

To the best of our knowledge, there are only two previous reports of a 3-mm stone being the smallest to cause such complications. Nedjim et al. were the first to report a spontaneous rupture of the fornix due to a 3-mm ureteral stone causing urinoma [15]. Khashan et al. described a similar case involving a 3-mm stone at the VUJ, causing obstructive uropathy and renal calyceal rupture in a 19-year-old female patient, with the patient being treated conservatively [11]. Our case represents the third instance of a 3-mm stone causing similar issues; however, due to persistent pain and the risk of worsening urinary extravasation, the patient underwent cystoscopy and JJ stenting after 24 hours.

## Conclusions

This case demonstrates how even small ureteric stones, like the 3-mm stone discussed here, can result in significant complications like spontaneous calyceal rupture. Early and accurate diagnosis using imaging, particularly contrast-enhanced CT, is critical in differentiating such cases from other potential causes of abdominal pain. While conservative management is often sufficient for small, passable stones, persistent symptoms and risk of complications may necessitate prompt surgical intervention, as demonstrated by the successful outcome following cystoscopy and JJ stenting in this patient. This case highlights the importance of vigilance in managing obstructive uropathy to prevent severe outcomes such as urosepsis, urinoma formation, or renal damage, emphasizing the need for tailored and timely therapeutic strategies.

## References

[1] Scales CD Jr, Smith AC, Hanley JM, Saigal CS (2012). Prevalence of kidney stones in the United States. Eur Urol.

[2] Leslie SW, Sajjad H, Murphy PB (2024). Renal calculi, nephrolithiasis. StatPearls [Internet].

[3] Chen GH, Hsiao PJ, Chang YH (2014). Spontaneous ureteral rupture and review of the literature. Am J Emerg Med.

[4] Assaker R, El Hasbani G, Thomas G, Sapire J, Kaye A (2020). Spontaneous rupture of the renal calyx secondary to a vesicoureteral junction calculus. Clin Imaging.

[5] Ashebu SD, Elshebiny YH, Dahniya MH (2000). Spontaneous rupture of the renal pelvis. Australas Radiol.

[6] Lien WC, Chen WJ, Wang HP, Liu KL, Hsu CC (2006). Spontaneous urinary extravasation: an overlooked cause of acute abdomen in ED. Am J Emerg Med.

[7] Worster A, Preyra I, Weaver B, Haines T (2002). The accuracy of noncontrast helical computed tomography versus intravenous pyelography in the diagnosis of suspected acute urolithiasis: a meta-analysis. Ann Emerg Med.

[8] Dym RJ, Duncan DR, Spektor M, Cohen HW, Scheinfeld MH (2014). Renal stones on portal venous phase contrast-enhanced CT: does intravenous contrast interfere with detection?. Abdom Imaging.

[9] Miller NL, Lingeman JE (2007). Management of kidney stones. BMJ.

[10] Wang H, Man LB, Huang GL, Li GZ, Wang JW (2016). Comparative efficacy of tamsulosin versus nifedipine for distal ureteral calculi: a meta-analysis. Drug Des Devel Ther.

[11] Khashan A, Kasanga S, Haq Z, Saini G, Talib S, Derbala S, Carson M (2023). Diminutive ureteral stone causing caylyceal rupture: case report and a review of the treatment options. Cureus.

[12] Al-Mujalhem AG, Aziz MS, Sultan MF, Al-Maghraby AM, Al-Shazly MA (2017). Spontaneous forniceal rupture: Can it be treated conservatively?. Urol Ann.

[13] Spinelli MG, Palmisano F, Zanetti SP (2019). Spontaneous upper urinary tract rupture caused by ureteric stones: a prospective high-volume single centre observational study and proposed management. Arch Esp Urol.

[14] Chaabouni A, Binous MY, Zakhama W, Chrayti H, Sfaxi M, Fodha M (2013). Spontaneous calyceal rupture caused by a ureteral calculus. Afr J Urol.

[15] Nedjim SA, Abdi M, Al Afifi M (2021). Spontaneous rupture of the fornix due to a ureteral lithiasis of 3 mm causing a urinoma: report of an original case. Radiol Case Rep.

